# Melatonin as an Alleviator in Decabromodiphenyl Ether-Induced Aberrant Hippocampal Neurogenesis and Synaptogenesis: The Role of Wnt7a

**DOI:** 10.3390/biom15081087

**Published:** 2025-07-27

**Authors:** Jinghua Shen, Lu Gao, Jingjing Gao, Licong Wang, Dongying Yan, Ying Wang, Jia Meng, Hong Li, Dawei Chen, Jie Wu

**Affiliations:** 1 Department of Occupational and Environmental Health, School of Public Health, Jinzhou Medical University, Jinzhou 121001, China; shenjh@stu.jzmu.edu.cn (J.S.); mengj@jzmu.edu.cn (J.M.); 2 School of Public Health, Jinzhou Medical University, Jinzhou 121001, China; 3 NHC Key Laboratory of Food Safety Risk Assessment, Chinese Academy of Medical Science Research Unit (No. 2019RU014), China National Center for Food Safety Risk Assessment, Beijing 100021, China

**Keywords:** decabromodiphenyl ether, melatonin, neurogenesis, synaptogenesis, Wnt7a

## Abstract

Developmental exposure to polybrominated diphenyl ethers (PBDEs), which are commonly used as flame retardants, results in irreversible cognitive impairments. Postnatal hippocampal neurogenesis, which occurs in the subgranular zone (SGZ) of the dentate gyrus, is critical for neuronal circuits and plasticity. Wnt7a-Frizzled5 (FZD5) is essential for both neurogenesis and synapse formation; moreover, Wnt signaling participates in PBDE neurotoxicity and also contributes to the neuroprotective effects of melatonin. Therefore, we investigated the impacts of perinatal decabromodiphenyl ether (BDE-209) exposure on hippocampal neurogenesis and synaptogenesis in juvenile rats through BrdU injection and Golgi staining, as well as the alleviation of melatonin pretreatment. Additionally, we identified the structural basis of Wnt7a and two compounds via molecular docking. The hippocampal neural progenitor pool (Sox2+BrdU+ and Sox2+GFAP+cells), immature neurons (DCX+) differentiated from neuroblasts, and the survival of mature neurons (NeuN+) in the dentate gyrus were inhibited. Moreover, in BDE-209-exposed offspring rats, it was observed that dendritic branching and spine density were reduced, alongside the long-lasting suppression of the Wnt7a-FZD5/β-catenin pathway and targeted genes (*Prox1*, *Neurod1*, *Neurogin2*, *Dlg4*, and *Netrin1*) expression. Melatonin alleviated BDE-209-disrupted memory, along with hippocampal neurogenesis and dendritogenesis, for which the restoration of Wnt7a-FZD5 signaling may be beneficial. This study suggested that melatonin could represent a potential intervention for the cognitive deficits induced by PBDEs.

## 1. Introduction

Polybrominated diphenyl ethers (PBDEs) are commonly added to textiles, plastics, and electronic products for flame retardation. Due to their environmental stability, propensity for bioaccumulation, and known developmental neurotoxicity, extensive research has been conducted on their underlying mechanisms [1]. The concentrations of PBDEs in indoor dust in various regions of China, as well as in human blood, show that BDE-209 (decabromodiphenyl ether, deca-BDE) is the dominant congener [2]. Perinatal exposure to PBDEs is associated with irreversible cognitive impairments in children and adults [3,4,5], and large-scale cohort and systematic reviews have concluded that PBDEs impact externalizing behaviors and intelligence. In addition, BDE-47, -99, and -209 affect learning and memory in rodents [6].

Neurodevelopment begins early in gestation, continues throughout the neonatal period, and even spans into adulthood, during which neural stem cells (NSCs) actively proliferate and differentiate into neurons and glia [7]. The dentate gyrus (DG) of the hippocampus is unique because of its functional role in spatial memory. Furthermore, adult neurogenesis only occurs in the subgranular zone (SGZ) and the subventricular zone (SVZ) [8], which are critical for neural circuits and functional plasticity throughout life [9]. Prenatal exposure to decaBDE (in gestation days 1–14, with 10, 30, or 50 mg/kg/day) impairs learning acquisition in male young rats (25 days-old) by affecting neurogenesis in the hippocampus during embryonic development, manifested as an inhibition in NSCs viability and differentiation [10], while the effects on postnatal hippocampal neurogenesis are still unclear.

Wnts are evolutionarily conserved secreted glycolipoproteins that regulate multiple aspects of hippocampal neurogenesis [11,12]. In the canonical Wnt/β-catenin pathway, the Wnt ligand interacts with the co-receptor low-density lipoprotein-related protein 5 or 6 (LRP5/6) to activate the Dishevelled (DVL) protein and induce the stabilization of β-catenin by preventing its phosphorylation by glycogen synthase kinase-3β (GSK-3β). Consequently, β-catenin accumulates in the cytoplasm and enters the nucleus, where it interacts with the transcription factors TCF/LEF to induce target gene transcription [13,14]. Among canonical Wnt molecules (Wnt1, Wnt3, Wnt7, and Wnt8), Wnt7a is critical for axonal guidance and dendritogenesis [15]. Wnt7a-knockout mice presented fewer neural progenitor cells (NPCs) and impaired morphology of hippocampal granule cells, such as decreased dendritic length and complexity [16]. In contrast, chronic hippocampal infusion of Wnt7a increased arborization and the number of newborn neurons [17]. Moreover, Wnt7a stimulates dendritic morphogenesis via GSK-3β inhibition, thus triggering β-catenin-TCF/LEF-dependent postsynaptic density-95 (PSD-95/*Dlg4*) transcription and protein expression [18].

Melatonin, N-acetyl-5-methoxytryptamine, a hormone secreted mainly by the pineal gland in the mammalian brain, plays an important role in neurogenesis [19]; for example, it promotes viability, proliferation, and neuronal differentiation in mouse NSCs [20], as well as dendritogenesis in adult rat hippocampal organotypic cultures [21]. Multiple signaling pathways are involved, including Wnt/β-catenin activation, as melatonin effectively rescues neonatal sevoflurane-induced Wnt suppression and alleviates long-term synaptic toxicity [22]. The neurotoxicity of BDE-209 is similar to BDE-47; according to RNA-seq analysis, both interfere with gene expression in the Wnt signaling pathway and influence axon guidance and neuron transmission-related processes, especially during early developmental stages [23,24]. Therefore, we investigated the impacts of perinatal BDE-209 exposure on hippocampal neurogenesis and synaptogenesis in juvenile rats, the role of the canonical Wnt7a pathway during these processes, and restoration with melatonin. Through this investigation, a potential intervention for the neurotoxicity of PBDEs was suggested.

## 2. Materials and Methods

### 2.1. Animals and Treatment

All animal experiments were approved by the Animal Ethics Committee of Jinzhou Medical University (approval number: 2023030) and complied with the National Institutes of Health Guide for the Care and Use of Laboratory Animals. Sprague Dawley rats aged 10–12 weeks (weighing 250 g ± 20 g) were obtained from the Changsheng Biotechnology (Liaoning, China, license number: SYXK 2020-0001) and were housed in standard conditions of temperature (22 ± 2 °C) and relative humidity (50 ± 5%) with access to food and water ad libitum under a 12/12 h light/dark cycle. After 1 week of acclimation, female and male rats were mated at a 2:1 ratio, and the day of vaginal plug appearance was recorded as gestation day 0. Pregnant rats were randomly divided into five groups and administered during pregnancy and lactation, except on the parturition day. BDE groups were treated daily with 30 or 100 mg/kg BDE-209 (5 mL/kg body weight by gavage; CAS#1163-19-5, J&K Scientific, Beijing, China). Melatonin (MT, CAS#73-31-4, Sigma, Shanghai, China) groups were subcutaneously injected with 10 mg/kg of melatonin 2 h before BDE-209 administration at 8–10 a.m., as previously described [25], and the MT+BDE209 group received a higher dose of BDE209 (100 mg/kg). Control group rats received saline or corn oil instead. The high dose 100 mg/kg was selected based on a previous report, at which point perinatal BDE-209 exposure induced significant alteration in neuronal migration of the fetal mouse cerebral cortex, while it did not affect pregnancy maintenance or delivery [26].

### 2.2. Novel Object Recognition (NOR)

The NOR apparatus included a square arena (72 cm × 72 cm) within a 40-lux light intensity testing room, as well as cameras and software to record behavior. The test consisted of three periods: first was adaptation, rats were gently placed in the arena to acclimate the experimental environment for 5 min; second was training, where two similar objects were placed in the arena with same interval to the sidewall, allowing rats to explore these objects freely for 5 min; third was testing, after a 1 h interval, one object was replaced to a new one with same material but different shapes, after which the rats were placed back to explore for 5 min. Exploration was defined as sniffing the object with the nose within 1 cm radius or touching. The apparatus was routinely wiped with 70% alcohol to eliminate the interference of biases and olfactory cues [27]. The recognition index (RI) = time exploring new object/(time exploring new object + time exploring old object). Higher RI indicates better recognition memory.

### 2.3. Morris Water Maze (WMW)

The MWM protocol includes a navigation training session and a probe trial. A 150 cm diameter circular tank was filled with water (22 ± 1 °C) that was made to be opaque. Animal swimming trajectories were recorded using a digital camera coupled with ANY-maze video tracking System (Stoelting Co., Wood Dale, IL, USA). During the learning task, a platform (10 cm in diameter) was placed in the middle of target quadrant 1 cm below the water surface. Rats were trained to find the platform via four 60 sec trials per day in a pseudorandom location order for 5 consecutive days. A trial was terminated once the rat found the hidden platform, and the average time spent was defined as the escape latency. The rats that failed to find the platform within 60 sec were manually guided to and allowed to rest on the platform for 15 sec. On day 6, a probe trial was conducted with the escape platform removed, and the number of platform crossings and time spent in the target quadrant was recorded to reflect the spatial memory of rats.

### 2.4. BrdU Staining

Thymidine analogue bromodeoxyuridine (BrdU, ≥99%, B5002, Sigma, Shanghai, China) dissolved in ethanol and diluted with saline was administered via intraperitoneal injection to rats. To evaluate the proliferation of NPCs in the hippocampus subgranular zone (SGZ), rats received a single dose of BrdU (50 mg/kg) on postnatal day 7 (P7) and sacrificed 4 h later. To assess neurons differentiated from NPCs, 3 pups randomly selected from 3 litters of each group were treated with 50 mg/kg BrdU once daily for 3 consecutive days from P7 to P9. They were sacrificed on P21 to assess neuroblast survival and immature neurons by co-immunostaining with BrdU and DCX.

### 2.5. Tissue Collection and Immunofluorescence

On PND7, PND21, and PD35, pups were deeply anaesthetized via intraperitoneal injection of 0.4% pentobarbital sodium and transcardially perfused with cold saline, followed by 4% paraformaldehyde for fixation. The brain tissues containing hippocampus were fixed in 4% paraformaldehyde for 48 h at 4 °C, transferred into 20% sucrose–phosphate buffer (PB) for 24 h, and then 30% sucrose–PB for another 48 h. They were finally embedded in Optimal Cutting Temperature Compound (OCT) and frozen at −80 °C. Then, 30 μm thick consecutive sections [28] of the dentate gyrus area of the hippocampus were cut using a cryostat microtome (Leica CM1950, Wetzlar, Germany).

For immunofluorescence, frozen sections were blocked with 5% goat serum-0.3% Triton X-100 for 1 h at room temperature, then incubated with primary antibodies overnight at 4 °C. The sections were subsequently incubated with secondary antibodies for 1 h in a moist and dark environment. The fluorescence signal was visualized using a fluorescence microscope (Leica M165FC, Wetzlar, Germany) and analyzed using ImageJ software v1.8.0.

For BrdU detection, frozen sections were incubated in 2 N HCl for 30 min at 37 °C to denature DNA, then washed with 0.01 M PBS 2 times for 5 min each. After these additional steps, immunofluorescence staining was performed with the blocking process as described above. The primary antibodies used were as follows: mouse anti-BrdU (1/100, A1482, ABclonal, Wuhan, China), rabbit anti-SOX2 (1/200, 11064-1-AP, Proteintech, Wuhan, China), rabbit anti-DCX (1/500, ab207175), mouse anti-Ki67 (1/500, ab279653), and rabbit anti-NeuN (1/500, ab177487, Abcam, Shanghai, China).

### 2.6. Golgi–Cox Staining

Golgi–Cox staining for the morphometry of neuronal dendrites was conducted according to the manufacturer’s instructions with the FD Rapid GolgiStain Kit (FD NeuroTechnologies, Ellicott City, MD, USA). After behavioral tests, pups were anesthetized, and brains were removed and quickly rinsed with ddH2O, then immersed in Golgi impregnation solution (a mixture of solution A and B at a ratio of 1:1) in the dark at room temperature for 2 weeks, then transferred into solution C for another 3 days. The brains were coronally sectioned at a 100 μm thickness with a cryostat microtome (Leica CM1950, Wetzlar, Germany) and placed onto gelatin-coated glass slides, then dried naturally in the dark. After being stained with solutions D and E, sections were dehydrated via gradient ethanol, cleared in xylene, and mounted with resinous medium. Secondary and tertiary apical dendrites in the DG area of the hippocampus were imaged using an Olympus microscope with a 100× oil immersion lens (BX53F, Tokyo, Japan), and dendritic arborization and density were analyzed using ImageJ software.

### 2.7. Western Blotting

Hippocampal tissue was homogenized in a RIPA buffer containing protease and phosphatase inhibitors, or in buffers according to the manufacturer’s instructions in the Nuclear and Cytoplasmic Protein Extraction Kit (#KGP150, Keygen Biotech, Nanjing, China). Proteins were separated on 8–15% gel via sodium dodecyl sulfate–polyacrylamide gel electrophoresis (SDS-PAGE) at a 120V voltage, then transferred onto polyvinylidene difluoride (PVDF) membranes at 100V for 60 min. After blocking with 5% nonfat dry milk or 5% BSA for 1 h at room temperature, membranes were incubated with primary antibody overnight at 4 °C, followed by incubation with HRP-conjugated goat anti-rabbit or anti-mouse IgG (1:5000, ABclonal, Wuhan, China), and finally exposed using a Chemiluminescence imaging system (General Electric, Chicago, IL, USA) using ECL substrate (Beyotime Biotech, Shanghai, China). Expression levels were quantified using ImageJ software and normalized to the internal reference of GAPDH (1:10,000) or PCNA (1:5000, Proteintech, Wuhan, China). Primary antibodies with dilution used were as follows: the phospho-β-catenin (Ser33/37/Thr41) antibody was from CST (#9561); rabbit polyclonal antibodies, β-catenin (1:5000, #51067-2-AP), PSD95 (1:2000), MAP2 (1:2000, #17490-1-AP), Prox1(1:1000, #11067-2-AP), and NeuroD1 (1:1000, #12081-1-AP) were from Proteintech (Wuhan, China); Neurog2 (#A19800), Netrin1 (#A16236), CRMP3 (#A17615), Dvl1 (#A10536), phospho-GSK3β-Ser9 (#AP0039), and GSK3β (#A2081, 1:1000) were from Abclonal (Wuhan, China); and Wnt3a (#sc-136163) was from Santa Cruz (Dallas, TX, USA).

### 2.8. Co-Immunoprecipitation

Total proteins of the hippocampus were extracted using NP-40 lysis buffer (Beyotime Biotech, Shanghai, China), and the interaction between Wnt7a with FZD5 was analyzed using the BeaverBeads^TM^ Protein A/G Immunoprecipitation Kit (Beaver #22202-100, Suzhou, China) according to the manufacturer’s instructions. Magnetic beads were bound with a 1 μg antibody (mouse anti-Wnt7a, Santa Cruz # sc-365459, Dallas, TX, USA) for 60 min at room temperature, washed using Binding Buffer, then incubated with hippocampal lysates on a rotator overnight at 4 °C. After washing using Washing Buffer, the antigen–antibody complexes were eluted using 1× SDS-PAGE loading buffer (Beyotime Biotech, Shanghai, China) and heated at 95 °C for 5 min. FZD5 binding to Wnt7a was determined with a Western blot using rabbit anti-FZD5 antibody (1:1000, Bioss # bs-2930R, Beijing, China).

### 2.9. Quantitative Real-Time PCR

Total RNA from hippocampus was extracted following the protocol for RNAiso Plus (Takara #9108, Dalian, China) and reverse-transcribed into cDNA using PrimeScript^TM^ RT reagent Kit (Takara #RR047A). QPCR was performed on an Applied Biosystems™ Real-Time PCR System (Thermo Scientific, Waltham, MA, USA) using TB Green Premix Ex Taq^TM^ II Kit (Takara #RR820A, Dalian, China) according to the manufacturer’s instructions, with primer sequences listed in Table 1. The running procedure was 1 cycle of 95 °C 30 s, 40 cycles of 95 °C 3 s and 60 °C 30 s, and then a melt curve stage. The relative mRNA level of target genes was calculated using the 2^−△△Ct^ method and normalized with Gapdh.

### 2.10. Molecular Docking

Molecular docking was performed to predict the binding affinity and interaction mechanisms between Wnt and melatonin, as well as BDE-209. The three-dimensional structures of Wnt7a (8TZP: pdb_00008tzp) and Wnt3a (8TZR: pdb_00008tzr) were obtained from the RCSB Protein Data Bank (https://www.rcsb.org/). The molecular structures of melatonin and BDE-209 were retrieved from the PubChem database (https://pubchem.ncbi.nlm.nih.gov (accessed on 3 June 2025)). Potential interactions were predicted using CB-DOCK2 (https://cadd.labshare.cn/cb-dock2/index.php), URL (accessed on 4 June 2025) [29], where the binding affinity was below −5.0 kcal/mol, indicating a stable binding capacity [30].

### 2.11. Statistical Analysis

Statistical analyses were performed via SPSS 26.0 (IBM SPSS, Amonk, NY, USA) and GraphPad Prism 8.0 (GraphPad, Boston, MA, USA). Two-way analysis of variance (ANOVA) with repeated measurements was used for the navigation test in the Morris water maze, and a two-way ANOVA was used for dendritic intersections. Statistical differences among groups were assessed using a one-way ANOVA followed by a post hoc test. Data were represented as mean ± standard deviation (SD), and *p* < 0.05 was considered statistically significant.

## 3. Results

### 3.1. Detailed Results

#### 3.1.1. Perinatal Melatonin Treatment Ameliorated BDE-209-Impaired Spatial Memory

**Figure 1:**
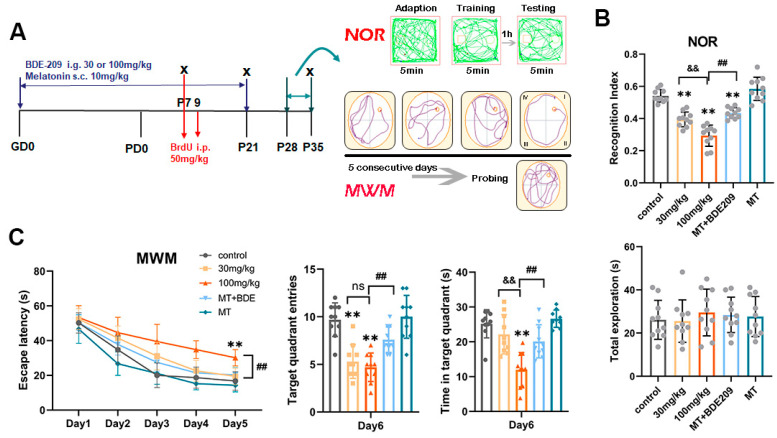
Melatonin ameliorated short-term and spatial memory in rats developmentally exposed to BDE-209. (**A**) Schematic of experimental design. Pregnant rats were subjected to BDE-209 intragastrically during pregnancy and lactation, with or without melatonin pretreatment via subcutaneous injection. BrdU was given at 50 mg/kg once on P7 or daily P7–P9 to detect proliferation of neural progenitor cells at P7 and neuronal differentiation at P21, respectively. Behavioral tests (Novel object recognition, NOR; Morris water maze, MWM) were conducted during P28–P35 to assess short- and long-term memory. (**B**) Recognition index for novel object and the total exploration times in NOR test. (**C**) Escape latency to reach the hidden platform during the navigation test in consecutive 5 days, platform crossings, and time spent in the target quadrant during the probe test. Data are presented as the mean ± SD (n = 10 rats per group). ** *p* < 0.01 vs. the control group; && *p* <  0.01 vs. 30 mg/kg group; ## *p* < 0.01 vs. 100 mg/kg BDE209 group; ns, no significance.

The NOR test was conducted to investigate short-term memory (Figure 1B). Total exploration time did not differ significantly among all the groups (F (4, 45) = 1.082, *p* = 0.377), while during the test trial, BDE-209-exposed rats spent significantly less time probing the novel object, thus the recognition index (RI) was obviously lower than that of the control (F (4, 45) = 47.394, *p* < 0.001). In contrast, melatonin-pretreated rats preferred to explore the novel rather than the familiar object (*p* < 0.001), indicating that melatonin prevented BDE-209-caused episodic memory impairment. Then, hippocampal-dependent spatial memory was evaluated using the Morris water maze. During navigation, the time taken to reach the hidden platform was gradually decreased with training days in all groups (Greenhouse–Geisser: F (time) = 187.819, *p* < 0.001; F (time*group interaction) = 1.937, *p* = 0.02), 100 mg/kg BDE-209-exposed rats exhibited prolonged latency on day 4 and day 5 compared to the control (both *p* < 0.001), and then during the day 6 probing, they crossed the hidden platform fewer times (F (4, 45) = 19.282, *p* < 0.001) and spent less time in the target quadrant (F (4, 45) = 16.398, *p* < 0.001), suggesting spatial memory deficit after BDE-209 exposure. Melatonin pretreatment restored these deficits, presented as a shorter escape latency (*p* = 0.001) and more crossings, together with a longer time spent in the goal quadrant (*p* crossings = 0.005, *p* time = 0.002) compared to the 100 mg/kg BDE209 group (Figure 1C).

#### 3.1.2. Melatonin Restored Hippocampal Neurogenesis in BDE-209-Exposed Young Rats

**Figure 2:**
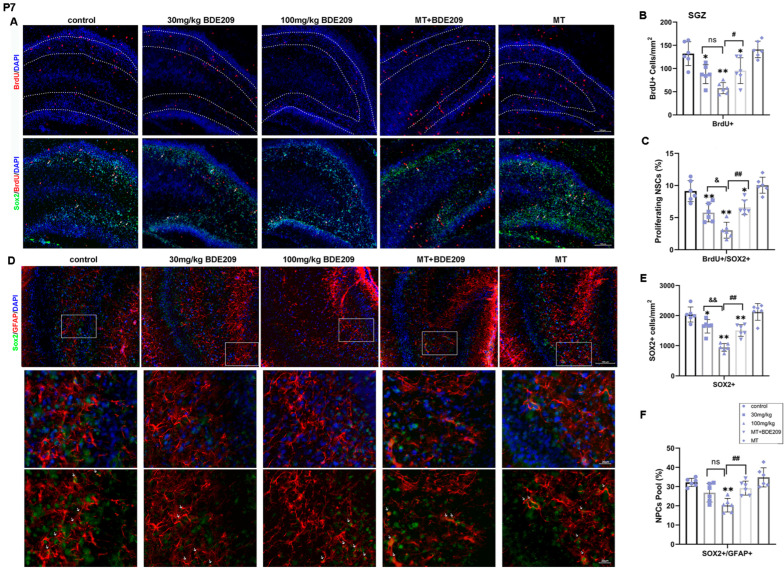
Perinatal melatonin treatment promoted proliferation of NPCs in hippocampal dentate gyrus (DG) of P7 BDE-209-exposed rats. (**A**) Representative images of BrdU (red), SOX2 (green), and DAPI (blue) in the DG of young rats. Scale bars, 100 μm. (**B**,**C**) Quantification of BrdU+ cells and proliferating NPCs (BrdU+SOX2+) in the SGZ. (**D**) Representative images of GFAP (red), SOX2 (green), and DAPI (blue) in the DG of young rats. Scale bars, 100 μm (upper), 20 μm (lower). (**E**,**F**) Quantification of NPCs (SOX2+) and NPC pool as SOX2+/GFAP+ cells in the SGZ. Data are presented as the mean ± SD (n = 6 images from 3 rats per group). * *p* < 0.05, ** *p* < 0.01 vs. the control; & *p* < 0.05, && *p* < 0.01 vs. 30 mg/kg group; # *p* < 0.05, ## *p* < 0.01 vs. 100 mg/kg BDE209 group. Neural progenitor cell, NPC. ns, no significance.

Hippocampal neurogenesis involves the process by which functional new neurons are generated from NSCs or NPCs during early development through adulthood, and it is essential for cognitive functions [31]. Transcription factor Sox2 is involved in the maintenance and regulation of NSC proliferation and fate determination, expressed in NSCs and putative NPCs [9]. Therefore, BrdU+ and Sox2+ cells were used to assess NPC proliferation in the DG of P7 rats (Figure 2A–C). ANOVA revealed that BDE-209 exposure decreased the number of BrdU+ cells (F (4, 25) = 15.09, *p* < 0.0001), as well as BrdU+/Sox2+ cells in SGZ (F (4, 25) = 25.57, *p* < 0.0001), indicating that the proliferation of NPCs was somehow inhibited. The potential of melatonin to modulate neurogenesis has been proposed in several preclinical studies, supported by in vivo evidence that exogenous melatonin administration increased NPC survival in the DG [31]. Here, pre- and postnatal melatonin pretreatment enhanced both BrdU+ cells (*p* = 0.012) and BrdU+/Sox2+ cells in SGZ (*p* = 0.001), compared with 100 mg/kg BDE-209-exposed rats, indicating the rescue of proliferating NPCs. Meanwhile, a significant reduction in Sox2+ cells in the DG was observed after BDE-209 exposure (F (4, 25) = 27.42, *p* < 0.0001), and the NPC pool, determined by the number of Sox2+/GFAP+ cells, was also markedly disrupted (F (4, 25) = 11.81, *p* < 0.0001). Melatonin resumed the NPC pool maintenance (*p* = 0.0055) and the number of Sox2+ cells (*p* = 0.0015) of 100 mg/kg BDE-209-exposed rats, while melatonin treatment alone exhibited no obvious effects versus the control (Figure 2D–F).

**Figure 3:**
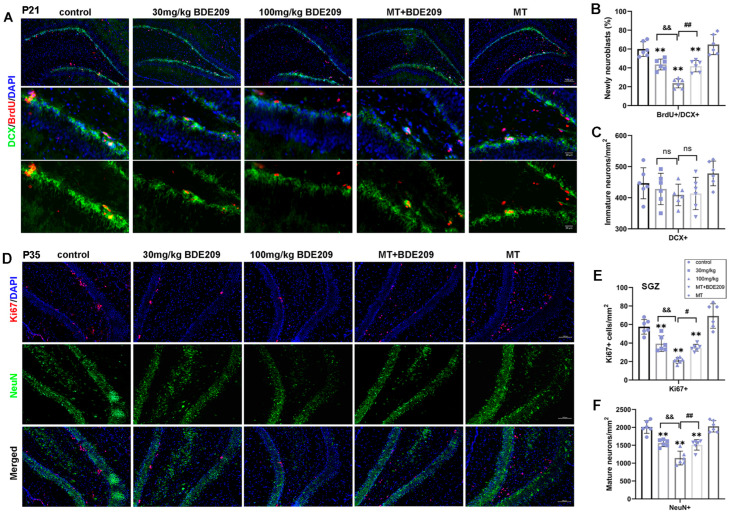
Melatonin treatment enhanced differentiation of NPCs into neurons and hippocampal adult neurogenesis of P35 BDE-209-exposed rats. (**A**) Representative images of BrdU (red), DCX (green), and DAPI (blue) in the DG of P21 rats. Scale bars, 100 μm (upper), 20 μm (lower). (**B**,**C**) Quantification of neuroblasts from NPCs (BrdU+DCX+) and immature neurons (DCX+) in the DG. (**D**) Representative images of Ki67 (red), NeuN (green), and DAPI (blue) in the DG of P35 rats. Scale bars, 100 μm. (**E**,**F**) Quantification of NPCs (Ki67+) in the SGZ, and mature neurons (NeuN+) in the DG. Data are presented as the mean ± SD (n = 6 images from 3 rats per group). ** *p* < 0.01 vs. the control; && *p* < 0.01 vs. 30 mg/kg group; # *p* < 0.05, ## *p* < 0.01 vs. 100 mg/kg BDE209 group. Neural progenitor cell, NPC. ns, no significance.

Doublecortin (DCX) is a protein present on immature neurons that has been used as a common marker of neurogenesis [32]. Thus, DCX+ and BrdU+ cells in the DG, considered as migrating neuroblasts or immature newborn neurons, were counted to investigate NPC differentiation at P21 (Figure 3A–C). A remarkable decrease in BrdU+/DCX+ cells (F (4, 25) = 29.74, *p* < 0.0001) was observed in hippocampal DG of perinatal BDE-209-exposed young rats, while not in DCX+ cells, although slightly decreased (P_DCX+_ > 0.05). Likewise, melatonin pretreatment promoted NPC differentiation into neurons, as shown by a significant increase in the number of BrdU+/DCX+ cells (*p* = 0.002) and DCX+ cells without significant alteration (P_DCX+_ > 0.05).

Adult neurogenesis occurs at approximately 4 weeks postnatal; during this synaptic integration, newly generated neurons establish their synaptic contacts into the pre-existing circuits [9]. Therefore, double staining for NeuN and Ki67 was performed to assess the maturity of neurons and the proliferation of NSCs in the hippocampal SGZ region of P35 rats (Figure 3D–F). There was a significant difference in the number of NeuN+ cells in the DG among all groups (F (4, 25) = 33.56, *p* < 0.0001), indicating that BDE-209 reduced mature neurons in the granular layer, partly attributed to earlier decline in the NPC pool and differentiation. Furthermore, Ki67+ immunostaining showed positive cells mainly concentrated in the SGZ of DG, which decreased significantly in young rats after BDE-209 exposure (F (4, 25) = 32.27, *p* < 0.0001). Both NeuN+ and Ki67+ cell counts were markedly restored in melatonin-treated rats (P_NeuN+_ = 0.0037, P_Ki67+_ = 0.0112), while rats that received melatonin alone showed no obvious enhancement over the control. These results suggested that melatonin rescued the survival of newly formed neurons and promoted NSC proliferation during adult neurogenesis in the hippocampus of BDE-209-exposed rats.

#### 3.1.3. Melatonin Ameliorated Impairments in Dendritic Branches and Spine Loss in BDE-209-Exposed Young Rats

**Figure 4:**
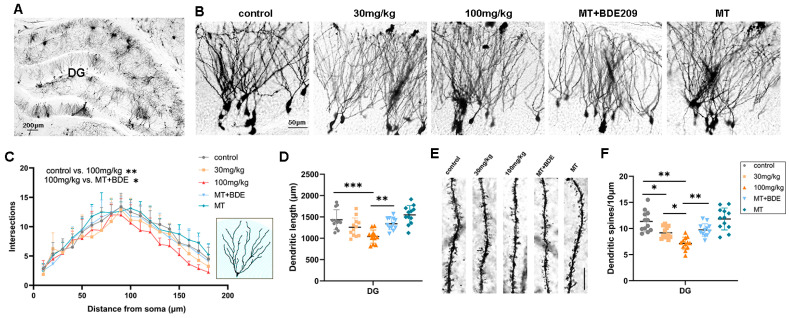
Melatonin enhanced dendritic arborization and spine density of granule neurons in hippocampal DG of P35 BDE-209-exposed rats. (**A**) Golgi staining of rat hippocampal DG and dentate gyrus. Scale bars, 200 μm. (**B**) Golgi staining of granule neurons of hippocampal DG region. Scale bars, 50 μm. (**C**,**D**) Dendritic intersections and total dendritic length (μm) of granule neurons in hippocampal DG, assessed using Sholl analysis. (**E**,**F**) Apical dendritic spine and density of hippocampal granule neurons, calculated as Spines/10 μm. Scale bars, 10 μm. n = 12 neurons from 3 rats per group. Data are presented as mean ± SD. * *p* < 0.05, ** *p* < 0.01, *** *p* < 0.001.

Structural and functional synaptic plasticity are widely considered as cellular mechanisms of learning and memory; therefore, dendritic arborization and the spine density of granule neurons in the hippocampal DG area were assessed. Golgi staining (Figure 4A,B) and Sholl analysis (Figure 4C,D) revealed a significant reduction in dendritic complexity in granule neurons of 100 mg/kg BDE-209-exposed rats (*p* = 0.0020 vs. control), as well as total dendritic length (*p* = 0.0127 vs. control), while melatonin demonstrated an obvious enhancement on both dendritic intersections (*p* = 0.0148) and total length (*p* = 0.0042) versus the 100 mg/kg BDE209 group under Turkey’s post hoc. Moreover, the apical dendritic spine density in granule neurons was also markedly decreased in BDE-209-exposed rats (P_30mg/kg_ = 0.0115, *p*_100mg/kg_ < 0.0001 vs. control), which was reversed by melatonin (*p* = 0.0018 vs. 100 mg/kg BDE209, Figure 4E,F), indicating alleviation of melatonin on BDE-209-induced synaptic hypogenesis in the hippocampal DG region of 5–6 week-old adolescent rats.

#### 3.1.4. Melatonin Triggered Canonical Wnt7a/β-Catenin Signaling via FZD5 Receptor in the Hippocampus of BDE-209-Exposed Young Rats

**Figure 5:**
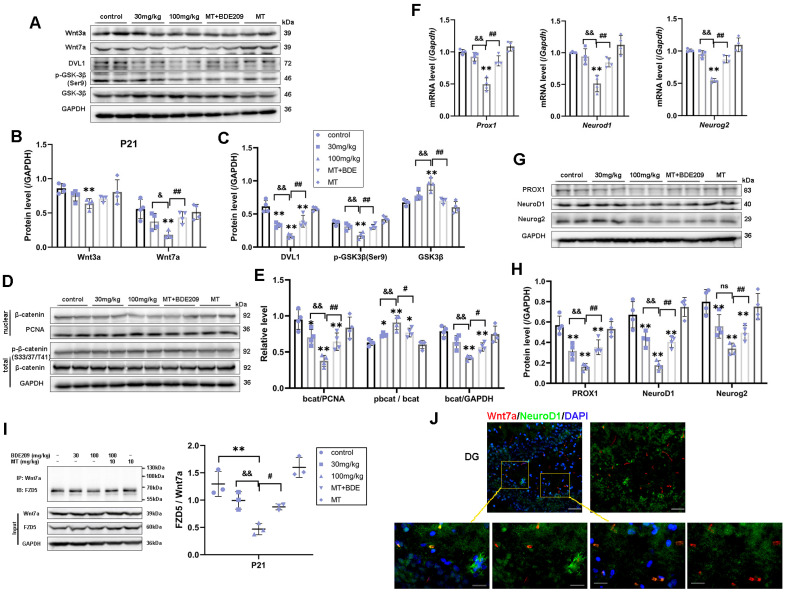
Wnt7a/FZD5-mediated canonical β-catenin-dependent genes thus promoted hippocampal neurogenesis in P21 rats. (**A**–**C**) Protein expression of Wnt3a, Wnt7a, DVL1, and downstream phos-GSK3β (Ser9) and total GSK3β. (**D**,**E**) Phosphorylated-β-catenin (Ser33/37/Thr41), total β-catenin, and nuclear translocation. (**F**) β-catenin target genes *Prox1*, *Neurod1*, and *Neurog2* transcriptional level. (**G**,**H**) Protein expression of Prox1, NeuroD1, and Neurog2. GAPDH and PCNA were used for internal reference of total protein and nuclear protein, respectively. (**I**) Co-immunoprecipitation analysis of FZD5 binding to Wnt7a in the hippocampus of P21 rats. (**J**) Immunofluorescence labeling for Wnt7a (Mu, Red), NeuroD1 (Rb, Green), and DAPI (blue) in hippocampal DG at P21 rats. Scale bar: upper—50 μm; lower—20 μm. Data are presented as the mean ± SD (n = 3–4 rats per group). * *p* < 0.05, ** *p* < 0.01 vs. the control; & *p* <  0.05, && *p* < 0.01 vs. 30 mg/kg group; # *p* < 0.05, ## *p* < 0.01 vs. 100 mg/kg BDE209 group. ns, no significance.

By activating Wnt/β-catenin signaling and specific downstream target genes, Wnt7a plays a critical role in multiple steps of neurogenesis, including NSC self-renewal, NPC proliferation, and neuronal differentiation and maturation [18]. Here, we examined whether the canonical Wnt7a/β-catenin signal was interrelated with hippocampal neurogenesis and synaptogenesis in juvenile rats after perinatal BDE-209 exposure and melatonin treatment. Firstly, reduced Wnt7a protein levels were observed in BDE-209-exposed rats at P21 (F (4, 15) = 7.831, *p* = 0.001), with a significant dose effect (r = −0.849, *p* < 0.001), as did the downstream proteins DVL1 (F (4, 15) = 34.771, *p* < 0.001) and phosphorylated GSK-3β (Ser9) (F (4, 15) = 18.785, *p* < 0.001). Consistent with Wnt7a ”off”, total GSK-3β and phosphorylation of β-catenin (Ser33/Ser37/Thr41) were significantly elevated in the 100 mg/kg BDE-209 group (P_GSK-3β_ = 0.001, Pp-bcat = 0.001), ultimately resulting in the proteasomal degradation of β-catenin (F (4, 15) = 13.978, *p* < 0.001) and the inhibition of nuclear translation (F (4, 15) = 12.950, *p* < 0.001). Thereafter, down-regulation in transcription of mRNA *Prox1*, *NeuroD1*, and *Neurog2* was shown in the 100 mg/kg BDE209 group (*p* < 0.001 vs. control), as well as corresponding protein expression of Prox1, NeuroD1, and Neurog2 (*p* < 0.01 vs. control). Melatonin recovered Wnt7a level (*p* = 0.004) and downstream molecules in the canonical pathway and subsequently elevated nuclear β-catenin (*p* = 0.007, Figure 5A–E), thus triggering Wnt target gene expression (post hoc test Pgenes < 0.01, Pproteins < 0.01 vs. 100 mg/kg BDE209, Figure 5F–H).

**Figure 6:**
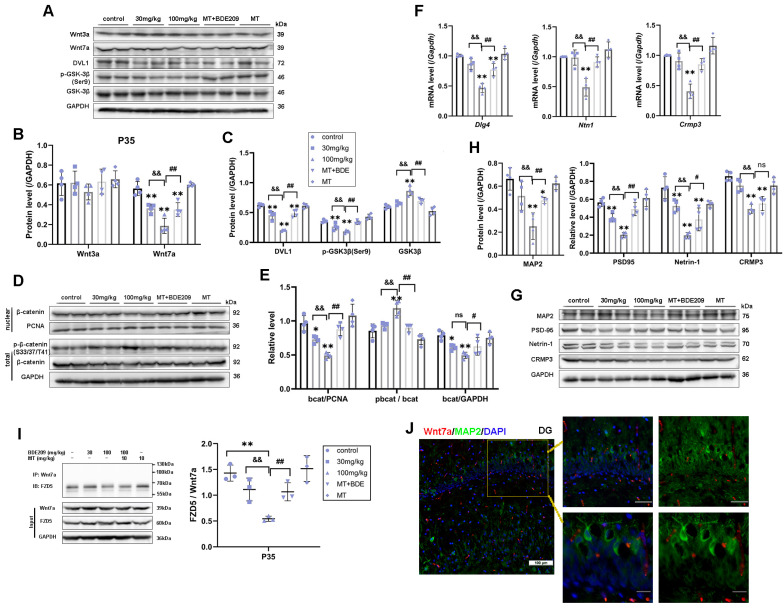
Melatonin triggered Wnt7a/FZD5 signaling target genes thus promoted synaptogenesis of P35 BDE-209-exposed rats. (**A**–**C**) Protein expression of Wnt3a, Wnt7a, DVL1, and downstream phos-GSK3β (Ser9) and total GSK3β. (**D**,**E**) Phos-β-catenin (Ser33/37/Thr41), total β-catenin, and nuclear translocation. (**F**) Wnt7a target genes *Dlg4*, *Nertin1*, and *Crmp3/Dpysl4* transcriptional level. (**G**,**H**) Protein expression of PSD95, Netrin1, CRMP3, and mature neurons’ marker MAP2. GAPDH and PCNA were used for internal reference of total protein and nuclear protein, respectively. (**I**) Co-immunoprecipitation analysis of FZD5 binding to Wnt7a in the hippocampus of P35 rats. (**J**) Immunofluorescence labeling for Wnt7a (Mu, Red), MAP2 (Rb, Green), and DAPI (blue) in hippocampal DG at P35 rats. Scale bar: upper—50 μm; lower—20 μm. Data are presented as the mean ± SD (n = 3–4 rats per group). * *p* < 0.05, ** *p* < 0.01 vs. the control; && *p* < 0.01 vs. 30 mg/kg group; # *p* < 0.05, ## *p* < 0.01 vs. 100 mg/kg BDE209 group. ns, no significance.

In addition to postnatal neurogenesis, Wnt also regulates synapse formation, neurotransmission, and plasticity [33]. Therefore, we further identified Wnt7a/β-catenin targeted genes for synaptogenesis and spine morphogenesis in the hippocampus of P35 rats. We found long-lasting Wnt7a canonical pathway inhibition in hippocampus of P35 rats after BDE-209 exposure, as ANOVA showed a significant decrease in the Wnt7a protein level (F (4, 15) = 12.418, *p* < 0.001), DVL1 protein level (F (4, 15) = 21.300, *p* < 0.001), and nuclear-β-catenin (F (4, 15) = 19.423, *p* < 0.001, Figure 6A–E). *Dlg4* (PSD-95) is a novel Wnt target gene essential for hippocampal synaptic plasticity [18]. Netrin-1, a secreted protein that is regulated by Wnt signaling, participates in axon guidance during nervous system development [34]. Collapsin response mediator protein 3 (CRMP3), also known as dihydropyrimidinase-related protein-4 (DRP-4, *DPYSL4*), is involved in dendritogenesis and regulated by the non-canonical Wnt pathway [35]. Both the transcription and translation of these genes were down-regulated in the hippocampus of 100 mg/kg BDE-209-exposed rats (*p* < 0.01); moreover, PSD-95 and Netrin-1 protein levels were also markedly decreased in the 30mg/kg BDE209 group (P_PSD95_ = 0.001, P_NTN1_ = 0.006, Figure 6F–H). Taken together, the canonical Wnt signaling, required for synapse function and stability, is affected in BDE-exposed rats. Similarly, melatonin restored Wnt7a/β-catenin (P_Wnt7a_ = 0.001, P_nu-bcat_ = 0.001), as did target genes and proteins for axon guidance, dendrite arborization, and spine maturation. Post hoc tests showed *p* < 0.01 vs. 100 mg/kg BDE-209 group, except for NTN1 (P_NTN1_ = 0.011) and CRMP3 (P_CRMP3_ = 0.232) proteins. Western blot original images can be found in Supplementary Materials.

**Figure 7:**
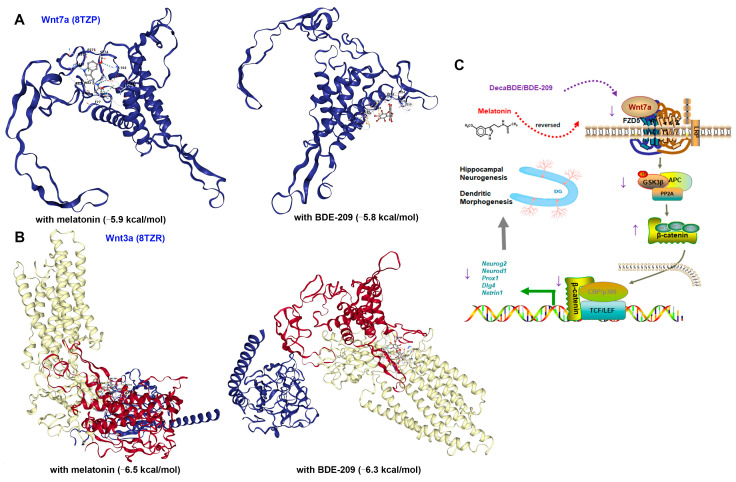
Molecular docking simulation and schematic mechanisms involved in this study. (**A**) Structure of melatonin and BDE-209 binding to Wnt7a. (**B**) Structure of melatonin and BDE-209 binding to Wnt3a. (**C**) Schematic illustration of canonical Wnt7a-FZD5/β-catenin signaling regulated hippocampal neurogenesis and synaptogenesis upon BDE-209 exposure and melatonin treatment. The schematic materials were sourced from ScienceSlides 2016 software and Figdraw platform (license number SOSASf4f9f).

Hippocampal stem/progenitor cells express receptors and signaling components for Wnt proteins, including Wnt3a, Wnt7a, and FZDs [12]. Since different Wnt/FZD ligand–receptor interactions are involved in the development of axons, dendrites, and synapses, numerous studies have focused on Wnt3a in developmental and adult neurogenesis [36]. Therefore, we concurrently measured Wnt3a in the hippocampus of juvenile rats. Marked down-regulation of Wnt3a protein was observed in 100 mg/kg BDE-209-treated rats at P21 (post hoc *p* = 0.007, Figure 5A,B), while not at P35 (Figure 6A,B), indicating that BDE-209-induced suppression of early postnatal neurogenesis additionally implicated Wnt3a, while synaptogenesis might mainly refer to Wnt7a/DVL signaling. Adjacently secreted Wnt7a of neurons was determined by immunofluorescent co-staining with NeuroD1 or MAP2, at P21 and P35, respectively, and both co-localization was observed (Figure 5J and Figure 6J). Considering the Wnt7a secretion mechanism depending on the molecular basis [37], we further analyzed the interaction between BDE-209/melatonin with Wnt using CB-DOCK2 (Figure 7A,B). Our data reveal that Wnt7a (PDB: 8TZP) exhibits stable binding affinities with both melatonin (−5.9 kcal/mol) and BDE-209 (−5.8 kcal/mol). For Wnt3a (PDB: 8TZR), these bindings are also notable, with a melatonin score of −6.5 kcal/mol, and BDE-209 at −6.3 kcal/mol, emphasizing the potential impacts on the Wnt signaling network.

Here, the question remains: which Frizzled receptor does Wnt7a signal through? The FZD5 receptor has been confirmed to mediate the synaptogenic effect of Wnt7a and promote presynaptic assembly through the canonical pathway [38,39]. We subsequently identified this interaction between FZD5 and Wnt7a via co-immunoprecipitation, as shown in Figure 5I and Figure 6I. After Wnt7a immunoprecipitation, the combined FZD5 level was markedly decreased in the hippocampus of 100 mg/kg BDE209-exposed rats both at P21 and P35 (*p* = 0.001 vs. control). Input results indicated that the hippocampal FZD5 protein was also reduced (F P21 = 15.281, *p* < 0.001; F P35 = 34.811, *p* < 0.001). Melatonin treatment promoted the combination of FZD5 with Wnt7a (P P21 = 0.011, P P35 = 0.006) and elevated the FZD5 protein level (P P21 = 0.002, P P35 = 0.002) compared to the 100 mg/kg BDE209 group. Overall, we concluded that Wnt7a/FZD5 signaling mainly regulated neuronal differentiation from NPCs and synapse formation through the canonical pathway, while non-canonical microtubule cytoskeletal components, like CRMP3, were also involved in the maintenance of dendritic spines and synaptic plasticity during postnatal neurodevelopment.

## 4. Discussion

Behavioral performance after maternal PBDE exposure is focused on cognition, like learning and memory, attention, and executive function [5]. In this study, we evaluated recognition and spatial memory deficits through NOR and MWM tests. BDE-209-exposed rats took longer to locate the hidden platform, crossed fewer quadrants, and spent less time in the target quadrant, in line with previous reports [40]. They also presented a reduction in the recognition index for novel objects without alteration in total explorations for old and new objects [41], suggesting abnormal function in the hippocampus. Postnatal neurogenesis is fundamental for neuronal plasticity [9], while disturbance in neurogenesis has been implicated in neurotoxicity of PBDEs: BDE-47 inhibited the proliferation and expansion of hESC-derived NPCs at sub-lethal concentrations [42]; developmental BDE-209 exposure (GD6~PD16) inhibited the proliferation of type-B stem cells (BrdU+GFAP+), and decreased SVZ-derived olfactory granule cells in offspring mice, thus impacting SVZ neurogenesis [26]. During hippocampal neurogenesis, NSCs existing in SGZ generate intermediate NPCs, which give rise to neuroblasts that differentiate into DG neurons [32]. Here, we assessed the hippocampal neurogenesis of offspring rats at PD7 for NSC/NPC proliferation (BrdU+Sox2+) and NPC pool (SOX2+GFAP+), at P21 for neuroblasts derived from NPCs (BrdU+DCX+) and immature neurons (DCX+), as well as at P35 for mature neurons (NeuN+) and adult neurogenesis in SGZ (Ki67+).

Identical to the previous in vitro study [43], the proliferation of hippocampal NSCs was inhibited after BDE-209 exposure; moreover, the NPC pool was disrupted, and new neuroblasts were correspondingly reduced. Consequently, immature neurons and mature neurons’ survival was reduced. Taken together, these findings indicate that irregular proliferation and differentiation processes in the SGZ contribute to BDE-209-induced memory deficits. In contrast to earlier reports, the number of newborn neuroblasts (BrdU+DCX+) increased in the olfactory bundle of P16 mice in the 100 mg/kg BDE-209 group [26], DCX expression was increased by 30 mg/kg decaBDE in the hippocampus of E13 mouse fetuses [44], and we have neither observed elevation in DCX+ cells nor DCX expression here, probably on account of different developmental stages or priority for glial differentiation. Since it is required for NPCs maintenance, Sox2 supports the proliferation and self-renewal of adult NSCs in the DG. Further exploration of adult neurogenesis should include co-staining of Ki67 or BrdU with Sox2, although Ki67+ cells here at SGZ of P35 rats most likely indicate NSC/NPC proliferation.

The geometry of the dendritic arbor directly determines synaptic density and the size of the receptive field, both of which influence the firing pattern of the neuron [45]. The canonical Wnt/β-catenin pathway regulates stem cell proliferation, cell fate determination, and synapse formation by modulating gene expression [11]. As reported, BDE-47 reduced the dendritic length and complexity of the branching pattern, as well as spine density in the offspring prefrontal cortex [46]. A reduction in dendritic branching and density of dendritic spines in the hippocampal DG region of perinatal BDE-209-exposed rats, as well as long-lasting suppression of Wnt7a/β-catenin signaling and targeted gene expression at P21 and P35, were observed in this study. Among them, pro-neural transcription factors Neurog2, NeuroD1, and Prox1 have been identified as targets of β-catenin-TCF/LEF signaling [9], as well as Netrin-1 and PSD95/Dlg4, which participate in axon guidance and dendritic spine morphogenesis. Moreover, CRMP3/Dpysl4 is involved in the non-canonical CaMKII pathway and has a profound influence on neurite initiation, dendritic complexity, and dendritic spine development, collectively affecting learning and memory [47].

Many Wnt ligands and Frizzled receptors, including Wnt7a/b, FZD5, FZD7, and co-receptor LRP5/6, are expressed in the postnatal and adult hippocampus [48]. Indeed, FZD5 and FZD7 are two receptors for Wnt7a, required for presynaptic assembly and postsynaptic plasticity, respectively, through different DVL pathways, like β-catenin or non-canonical Ca^2+^/CaMKII [38]. In hippocampal neurons, Wnt7a-FZD5 is required for the formation of pre-synaptic sites, while Wnt3a-FZD1 regulates pre-synaptic protein clustering and vesicle recycling [33]. Although both Wnt7a and Wnt3a were reduced in the hippocampus of P21 rats under BDE-209 exposure, only Wnt7a significantly decreased at P35, indicating that BDE-209-induced suppression of early postnatal neurogenesis was additionally implicated by Wnt3a, while synaptogenesis might mainly refer to Wnt7a signaling. Furthermore, interaction between FZD5 and Wnt7a presented a decreasing tendency, corresponding to downstream β-catenin pathway factors. Considering the postsynaptic assembly, FZD7 is a receptor for Wnt7a and Wnt7b and localizes to dendritic spines, regulating synaptic plasticity and promoting dendrite development through CaMKII activation [49,50]. Here, we observed that CRMP3/Dpysl4 was altered in P35 rats, and we speculated that the FZD5/7-mediated non-canonical CaMKII pathway may also be involved.

Melatonin, as a bioactive substance stimulating neurogenesis, normalized the proliferation of neonatal NPCs and differentiation-derived neuroblasts and increased dendritic arborization and spine density. It also stimulated the self-renewal of NSCs during adult neurogenesis in the DG of the hippocampus and, consequently, rescued BDE-209-caused memory impairment. As for Wnt activation, a previous report identified a structural inhibitor for the Wnt deacylase notum of melatonin [51]. Additionally, the MT1 receptor co-expressed well with β-catenin and Axin2 and bound to β-catenin by its C-terminal [22]. We further analyzed the docking affinity between Wnt7a/3a and melatonin/BDE-209 based on molecular structures associated with the biogenesis and secretion of Wnt. Our findings demonstrated robust interactions between Wnt7a (8TZP) and Wnt3a (8TZR) with these compounds. However, beyond Wnt/β-catenin, melatonin modulates several neurogenesis-related molecular mechanisms, e.g., BDNF-TrkB, Ca^2+^/CaMKII, and MEK/ERK1/2, as well as decreases autophagy and activates the mechanistic target of the rapamycin (mTOR) signaling pathway [52]. Therefore, the specific mechanism of melatonin on canonical Wnt7a/β-catenin signaling activation should be further determined.

## 5. Conclusions

In conclusion, developmental exposure to BDE-209 led to inhibition of hippocampal NPCs’ proliferation, neuronal differentiation, and synaptogenesis in the DG region of juvenile rats. Mechanically, the canonical Wnt7a/β-catenin pathway was primarily involved through the FZD5 receptor. Melatonin alleviated BDE-209-impaired hippocampal neurogenesis and dendritogenesis, in which restoration of Wnt7a-FZD5 signaling may be beneficial.

## Figures and Tables

**Table 1:** Primers used for real-time PCR.

Gene	Accession No.	Primer Sequences (5′-3′)
*Prox1*	NM_001107201	F: -GATGGCTCGTTTGCACATGG-
		R: -GGGTCAAACATCAGCTTCTGGAGTA-
*Neurod1*	NM_019218	F: -AAAGGTTTGTCCCAGCCCACTAC-
		R: -GCATGTCCGGATTCTGCTCA-
*Neurog2*	NM_001398677	F: -AGGCTGTGGGAATTTCACCTG-
		R:-GGGACAATAGGCATTGTGACGA-
*Dlg4*	NM_019621	F: -ACTGCATCCTTGCGAAGCAAC-
		R: -CGTCAATGACATGAAGCACATCC-
*Ntn1*	NM_053731	F: -TGCCAAAGGCTACCAGCAGA-
		R: -GAAGCCTTGCAGTAGGAGTCACAG-
*Crmp3*	NM_012933	F: -TGGGCTCTGATGCTGACCTG-
		R: -CACCACTCTGCCCTGACTTATGAC-
*Gapdh*	NM_017008	F: -GGCACAGTCAAGGCTGAGAATG-
		R: -ATGGTGGTGAAGACGCCAGTA-

## Data Availability

The original contributions presented in this study are included in the article/Supplementary Material, approval date (6 June 2025). Further inquiries can be directed to the corresponding author(s).

## Supplementary Materials

The following supporting information can be downloaded at: https://www.mdpi.com/article/10.3390/biom15081087/s1, Western blot original images.

## Abbreviations

The following abbreviations are used in this manuscript:

AMPAR	α-amino-3-hydroxy-5-methyl-4-isoxazolepropionic acid receptors.
BDE-209	Decabromodiphenyl ether.
BrdU	Bromodeoxyuridine.
CaMKII	Ca^2+^/CaM-dependent protein kinase 2.
CRMP3	Collapsin response mediator protein 3.
DG	Dentate gyrus.
DVL1	Dishevelled-1.
FZD	Frizzled.
GSK3β	Glycogen synthase kinase-3β.
MAP2	Microtubule-associated protein 2.
MT	Melatonin.
MWM	Morris water maze.
NeuroD1	Neurogenic differentiation factor 1.
Neurog2	Neurogenin-2.
NOR	Novel object recognition.
NPCs	Neural progenitor cells.
NSCs	Neural stem cells.
Prox1	Prospero homeobox protein 1.
SGZ	Subgranular zone.
SVZ	Subventricular zone.
PBDEs	Polybrominated diphenyl ethers.
PSD95	Postsynaptic density protein 95.
