# Supplementary material for: Melatonin as an Alleviator in Decabromodiphenyl Ether-Induced Aberrant Hippocampal Neurogenesis and Synaptogenesis: The Role of Wnt7a
Source: Biomolecules. 2025 Jul 27;15(8):1087. doi: 10.3390/biom15081087 (PMC12383937; doi:10.3390/biom15081087)

Figure 5A :

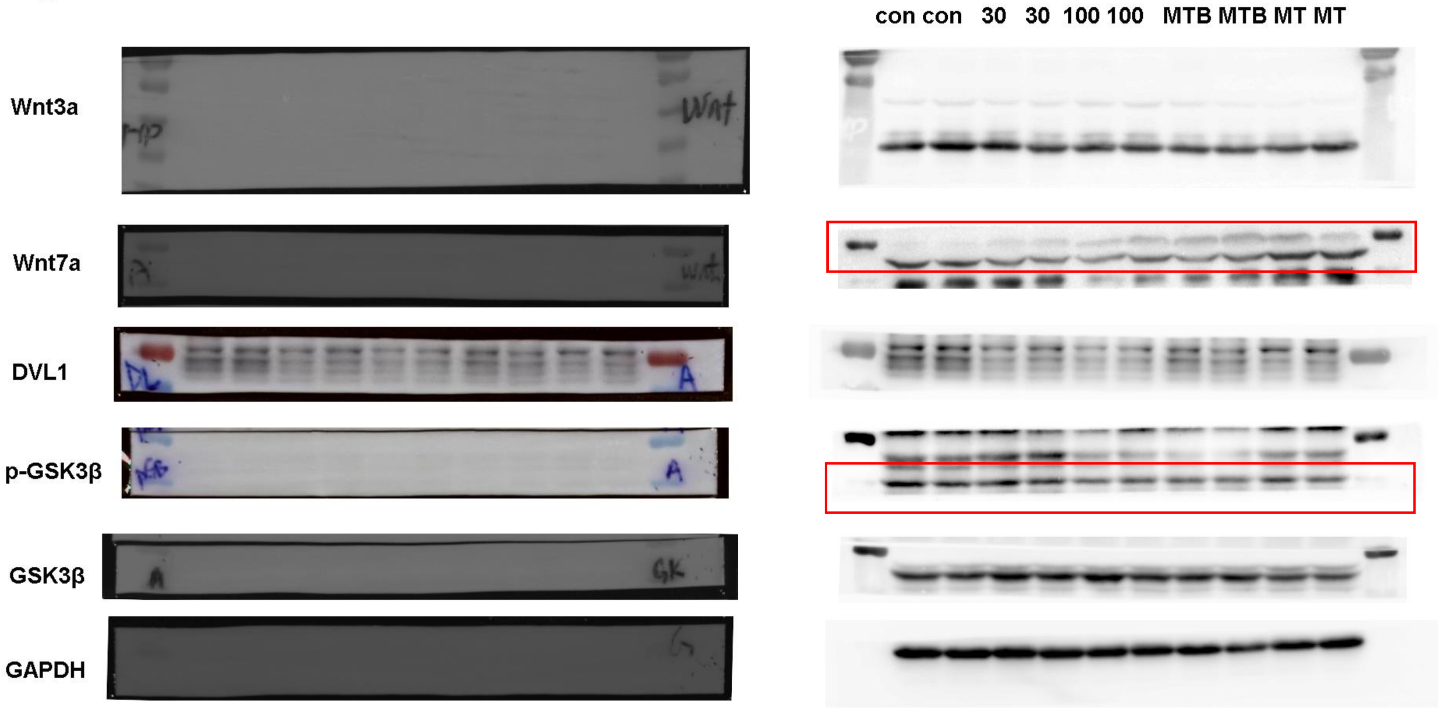
con con 30 30 100 100 MTB MTB MT MT
Wnt3a
Wnt7a
DVL1
p-GSK3β
GSK3β
GAPDH

Figure5D

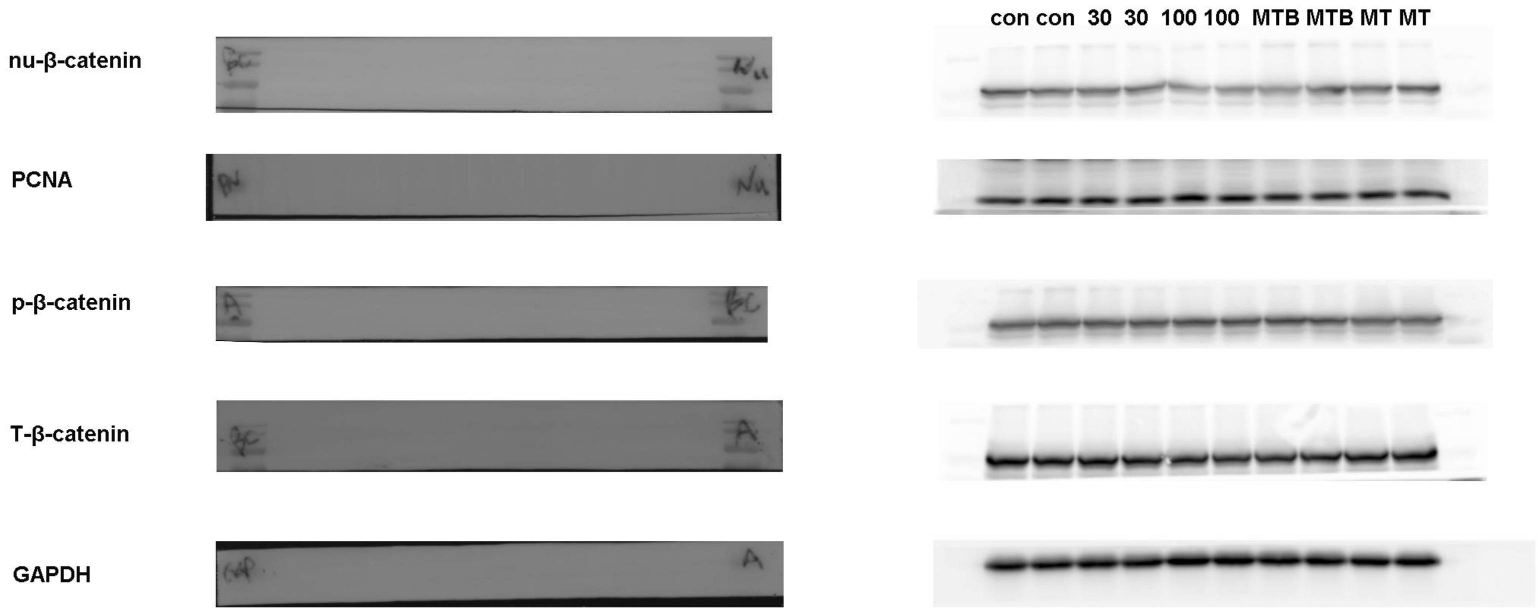
con con 30 30 100 100 MTB MTB MT MT
nu-β-catenin
PCNA
p-β-catenin
T-β-catenin
GAPDH

Figure 5G

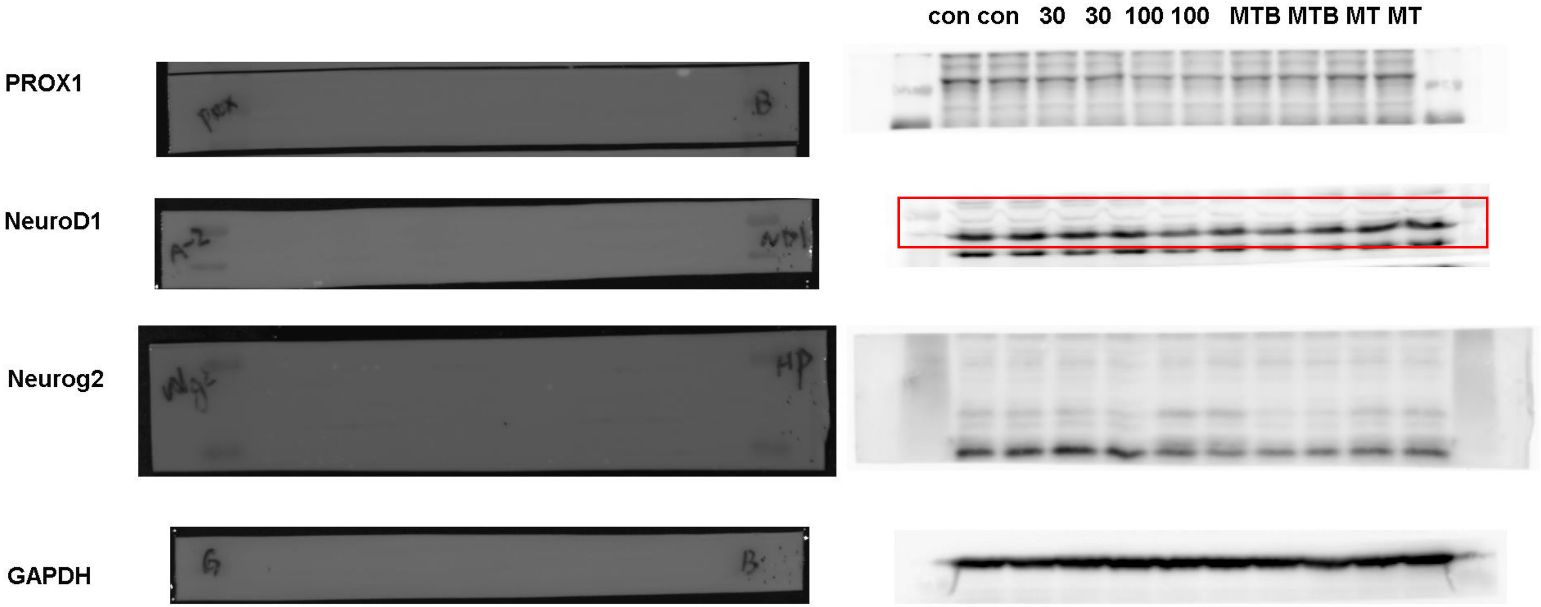
con con 30 30 100 100 MTB MTB MT MT
PROX1
NeuroD1
Neurog2
GAPDH

Figure 6A :

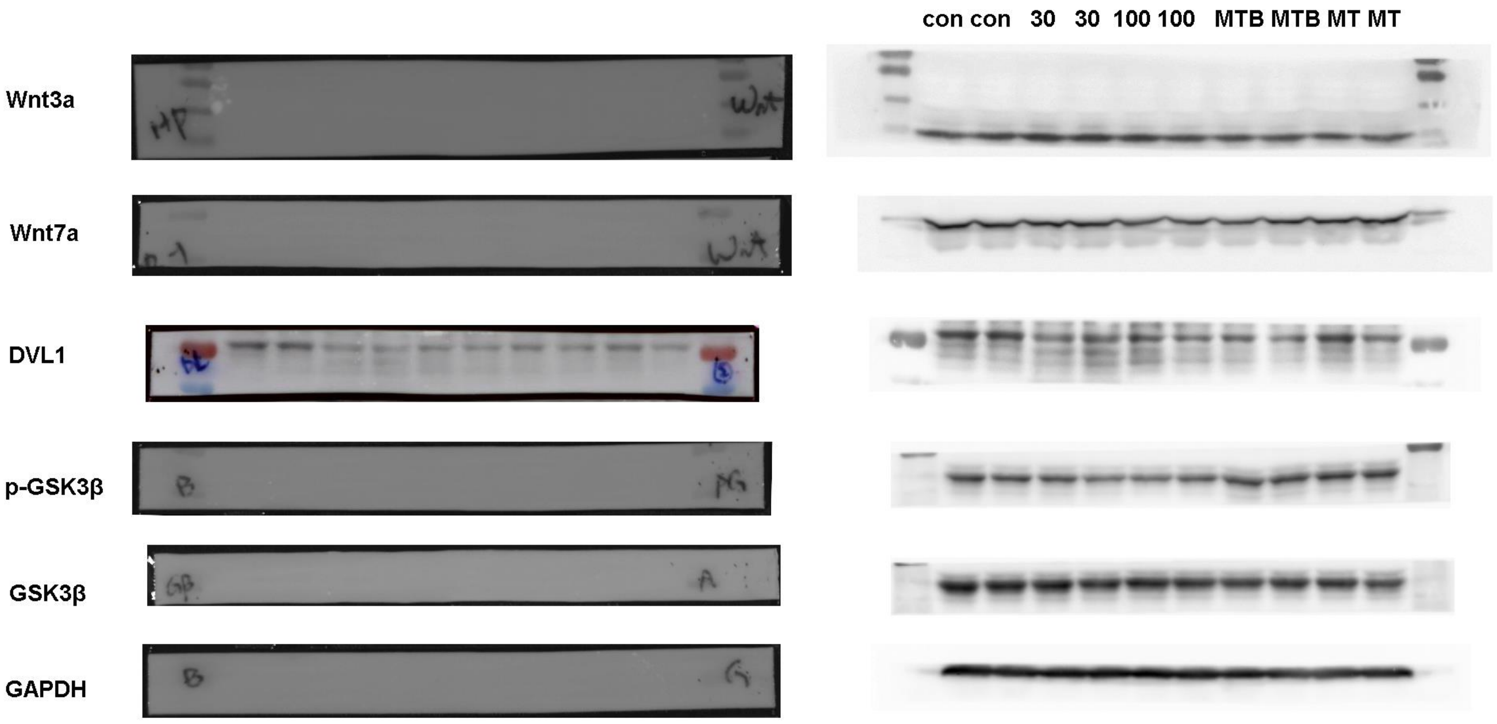

con con 30 30 100 100 MTB MTB MT MT
Wnt3a
Wnt7a
DVL1
p-GSK3β
GSK3β
GAPDH

Figure6D

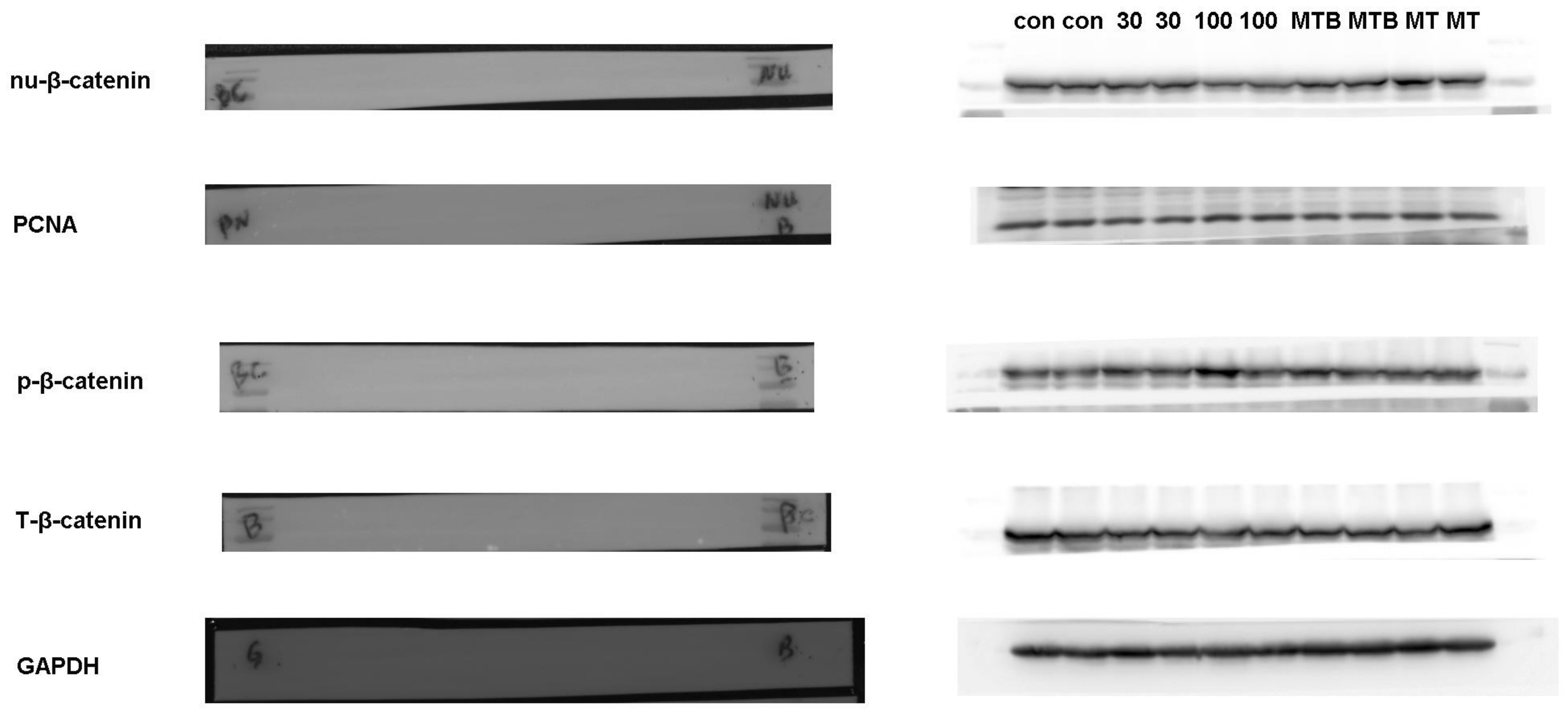
con con 30 30 100 100 MTB MTB MT MT
nu-β-catenin
PCNA
p-β-catenin
T-β-catenin
GAPDH

Figure 6G

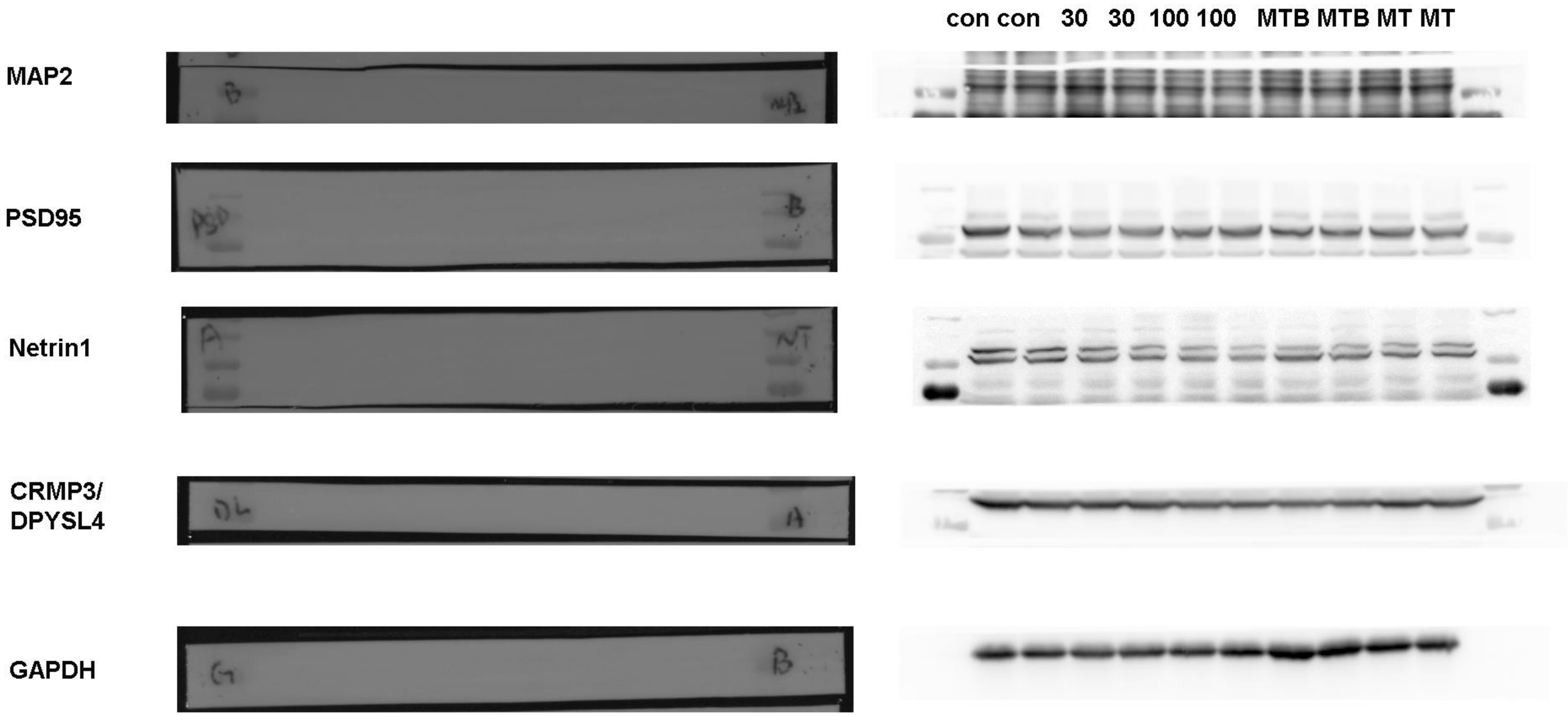
con con 30 30 100 100 MTB MTB MT MT
MAP2
PSD95
Netrin1
CRMP3/
DPYSL4
GAPDH

Figure5-I

IP: Wnt7a
IB: FZD5

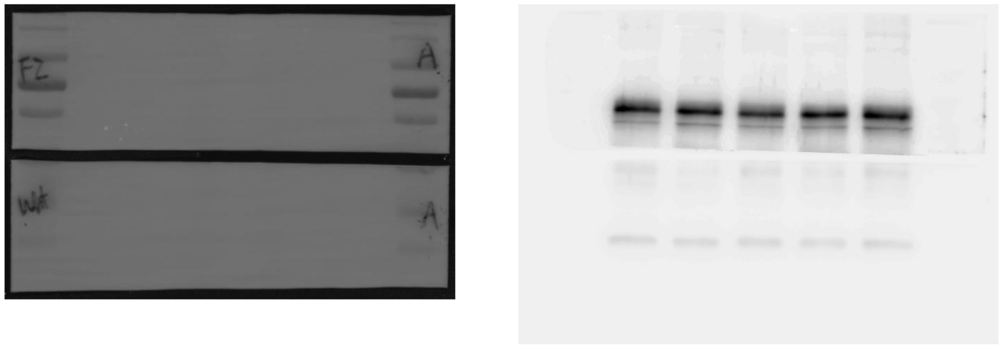

Input:

Wnt7a

FZD5

GAPDH

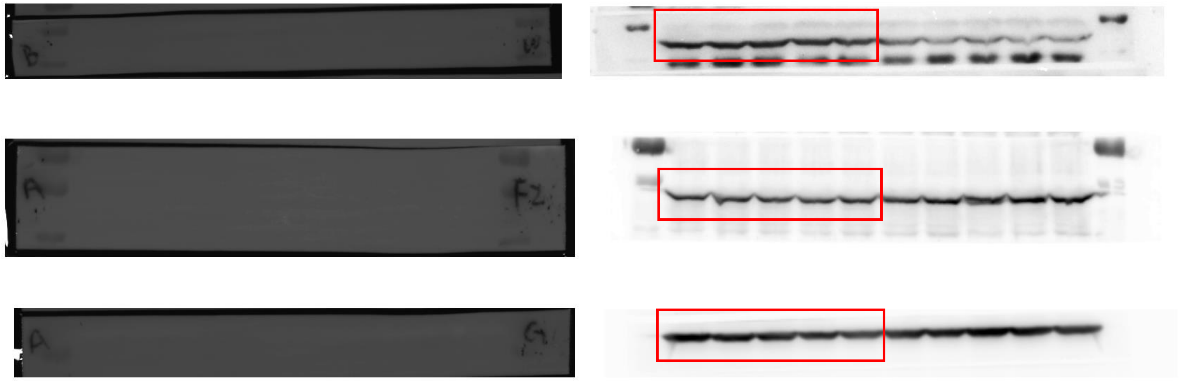

Figure6-I

IP: Wnt7a
IB: FZD5

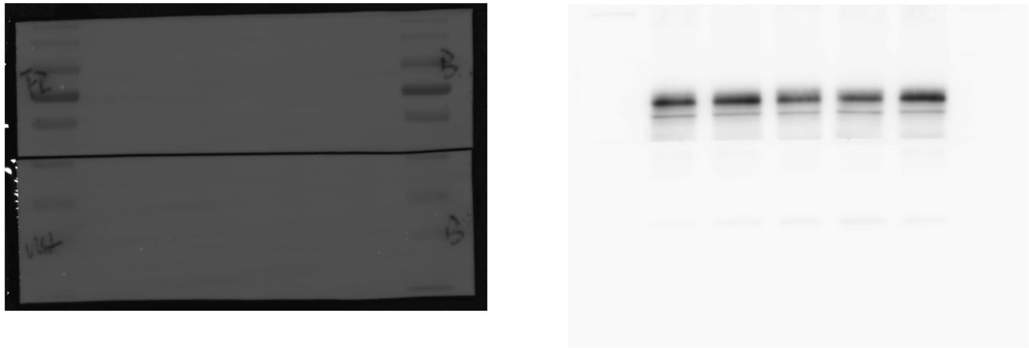

Input:

Wnt7a

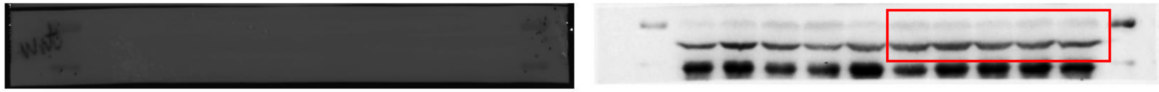

FZD5

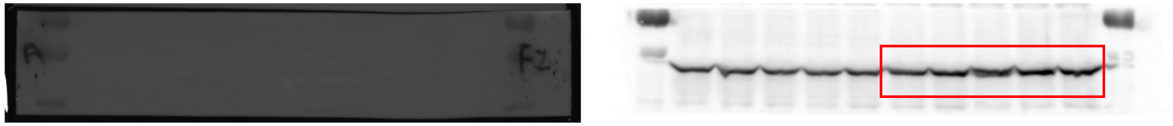

GAPDH

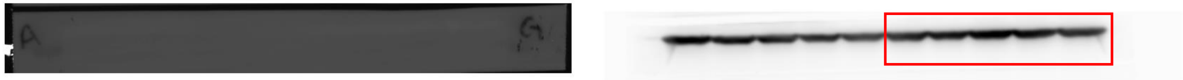

Supplement: Supplementary file 1 [file biomolecules-15-01087-s001.zip › biomolecules-3715713-supplementary.pdf]
